# Prediction of the Immune Phenotypes of Bladder Cancer Patients for Precision Oncology

**DOI:** 10.1109/OJEMB.2022.3163533

**Published:** 2022-04-15

**Authors:** Hyuna Cho, Feng Tong, Sungyong You, Sungyoung Jung, Won Hwa Kim, Jayoung Kim

**Affiliations:** ^1^ Graduate School of Artificial Intelligence (GSAI)Pohang University of Science and Technology 34995 Pohang 37673 South Korea; ^2^ Department of Computer Science and EngineeringUniversity of Texas at Arlington 12329 Arlington TX 76019 USA; ^3^ Department of Surgery and Biomedical SciencesCedars-Sinai Medical Center 22494 Los Angeles CA 90048 USA; ^4^ Department of Electrical EngineeringUniversity of Texas at Arlington 12329 Arlington TX 76019 USA; ^5^ Department of Computer Science and EngineeringPohang University of Science and Technology 34995 Pohang 37673 South Korea; ^6^ Department of MedicineUniversity of California Los Angeles 8783 Los Angeles CA 90095 USA

**Keywords:** Artificial algorithm, biomarker, bladder cancer, gene expression, immunotherapy, machine learning

## Abstract

Bladder cancer (BC) is the most common urinary malignancy; however accurate diagnosis and prediction of recurrence after therapies remain elusive. This study aimed to develop a biosignature of immunotherapy-based responses using gene expression data. Publicly available BC datasets were collected, and machine learning (ML) approaches were applied to identify a novel biosignature to differentiate patient subgroups. Immune phenotyping of BC in the IMvigor210 dataset included three subtypes: inflamed, excluded, and desert immune. Immune phenotypes were analyzed with gene expressions using traditional but powerful classification methods such as random forests, Deep Neural Networks (DNN), Support Vector Machines (SVM) together with boosting and feature selection methods. Specifically, DNN yielded the highest area under the curve (AUC) with precision and recall (PR) curves and receiver operating characteristic (ROC) curves for each phenotype ($0.711 \pm 0.092$ and $0.86 \pm 0.039$, respectively) resulting in the identification of gene expression features useful for immune phenotype classification. Our results suggest significant potential to further develop and utilize machine learning algorithms for analysis of BC and its precaution. In conclusion, the findings from this study present a novel gene expression assay that can accurately discriminate BC patients from controls. Upon further validation in independent cohorts, this gene signature could be developed into a predictive test that can support clinical evaluation and patient care.

## I. Introduction

Globally, bladder cancer (BC) is the ninth most common malignant tumor. BC also accounts for 4% of all cancer-related deaths in the United States, ranking it the fifth most deadly cancer [1]. According to the American Cancer Society, there will be approximately 83730 new cases of BC (about 64280 in men and 19450 in women) and about 17200 BC-related deaths (about 12260 in men and 4940 in women) in the United States, alone, in 2021. If your paper is intended for a conference, please contact your conference editor concerning acceptable word processor formats for your particular conference.

Based on the degree of bladder muscle wall infiltration, BC can be classified as either non-muscle invasive (NMIBC) or muscle invasive (MIBC). About 70% of BC patients have NMIBC, while the other 30% have MIBC or metastatic disease [2]. Treatment for NMIBC includes endoscopic resection of the tumor followed by adjuvant intravesical treatment to reduce the possibility of recurrence or progression. The risk of recurrence and progression is affected by many factors, including tumor grade, size, staging, multiplicity, recurrence rate, and the presence of carcinoma in situ (CIS). BC requires a lifetime of close monitoring and repeated treatments, which places an immensely heavy burden on patients and the social economy. MIBC treatment options include chemotherapy and radical cystectomy. The 5-year and 10-year survival rates of MIBC are approximately 50% and 36%, respectively. However, the 5-year survival rate of metastatic BC is only 15%, and the median overall survival (OS) is about 15 months following platinum-based chemotherapy.

Immunotherapies against BC have shown encouraging results. The first immunotherapy against BC was reported in 1976, when Alvaro Morales reported 9 cases of BC that were successfully treated with Bacillus Calmette-Guerin (BCG), demonstrating the immunogenicity of BC [3]. Immune checkpoint inhibitors (CPIs) are leading the field of immunotherapies against BC. It includes anti-cytotoxic T lymphocyte antigen 4 (CTLA4), anti-programmed cell death 1 (PD-1), and anti-programmed cell death 1 ligand 1 (PD-L1) antibodies. Anti-CTLA4, anti-PD-1, and anti-PD-L1 CPIs can improve anti-tumor immune response by restoring T-lymphocyte activation [4]. With the rapid advancement of new immunotherapy drugs, the development and validation of biomarkers will be important. Established biomarkers can help clinicians predict whether treatments will be effective. Varying subtypes of BC may also have definitive biological differences, which can result in variable sensitivity to Immunotherapies. In order to fully optimize the benefits of immunotherapy in future treatments and to further improve its impacts, supplemental biomarkers capable of monitoring response should be integrated.

Despite the initial success of cancer immunotherapies [5], approximately 70% of patients with advanced urethral cancer are considered unresponsive to anti-PD-1 or anti-PD-L1 antibodies [6], [7].

Recent studies have employed a variety of biomarkers such as PD-L1 hyperexpression and tumor mutation burden (TMB) to distinguish the potential immunotherapy responders from non-responders [5]. There seems to exist a link between these biomarkers and immunotherapy outcomes, but neither PD-L1 expression nor TMB was sufficient to distinguish immunotherapy responder from non-responders [8], [9]. For example, the epithelial PD-L1 expression in BC has been shown to be unrelated to immunotherapy responses [10]. In addition, there has been difficulty predicting responses using TMB as a single marker [11], although increased TMB has been linked to improved clinical outcomes of immunotherapy in bladder cancer [12]. These previous works indicate the unmet needs to identify more reliable biomarkers for the stratification of immunotherapy responders from non-responders.

IMvigor210 was an open multicenter, single-arm phase 2 clinical study designed to study whether atezolizumab could become the standard treatment for advanced urothelial cancer. This study suggested that for patients with first-line platinum-based refractory metastatic urothelial carcinoma (mUC) checkpoint inhibitors seem to be more attractive than chemotherapy [13]. Atezolizumab is now suggested to prescribe for many patients who are ineligible for cisplatin therapy. In our study we used the publicly available IMvigor210 data. Previously, IMvigor210 data has been used to test the prognostic power of gene expression signatures for basal and luminal/differentiated BC subtypes [14]. Overall survival, prognosis and response to immunotherapy were also studies in the IMvigor 210 cohort [15]. A consensus molecular classification system for MIBC was suggested by analyzing the 1750 MIBC transcriptomic profiles from datasets including IMvigor dataset, providing a tool for testing and validation of potential predictive MIBC biomarkers [16].

Big data-based ML has been increasingly used and successfully applied to preventive medicine, image recognition, diagnosis, personalized medicine, and clinical decision-making. Application of machine learning (ML) algorithms to determine the cancer-specific classifiers have been tried in a series of studies. To determine the multi-variate classifiers predicting response to paclitaxel-therapy, methylome and miRNome were used [16]. Not only in vivo multi-omics profiles [17] but also in vivo cancer molecular profiles were able to predict the drug-sensitive tumors using ML modeling approach [18].

Clinical application of conventional ML approaches has been performed for the more accurate clinical decision, which was benefited by an increased computational power and accumulated digital health data from patients [19], [20]. However, we are aware the limitations due to the complicated data processing (feature engineering) including knowledge-based training [21], [22]. ML algorithms derived from not-so-relevant data resources, low volume of patients, data with high sparsity and poor could significantly diminish enthusiasm and reduce the efficacy of ML approach [23].

Although ML is widely used in the context of BC, there are still limitations, including difficulties in quantitatively analyzing observed endpoints and the inapplicability of generalizability across data sets. Therefore, further verification is needed to improve the accuracy and versatility of ML in BC. Therefore, in this study, we aimed to search for the potential of using ML algorithms to investigate relationships between gene expression features with immunotherapies specific to BC and identify potentials to develop and use ML algorithms for such studies. For this, we have adopted five different traditional but powerful ML classification methods (i.e., Random Forest, Deep Neural Network, Support Vector Machine, Adaboost and XGBoost) to predict BC immune phenotypes using high-dimensional gene features. With efforts to avoid pitfalls of these algorithms, e.g., overfitting, we managed to get successful classification performance identifying phenotype-specific gene features (see Section IV for detailed clinical and technical discussions). We see great possibility to further develop more sophisticated and task-specific ML algorithm for analyzing BC with gene data to provide diagnostic tool for individuals and identify BC in their early stages, or possibly even prevent the disease.

## II. Materials and Methods

### A. Ethics Statement

For this paper, we used deposited datasets derived from previously published studies. Use of publicly deposited data does not require IRB approval.

### B. Description of the Dataset

For this study, we have used the Imvigor210 data that can be found in previous report [24] and the associated resource web site provided by Dorothee Nickles, Yasin Senbabaoglu, Daniel Sheinson at http://research-pub.gene.com/IMvigor210CoreBiologies/. The raw data are available at the European Genome-phenome archive (EGA) under the accession number EGAS00001002556. The IMvigor210CoreBiologies package can be downloaded at http://research-pub.gene.com/IMvigor210CoreBiologies/IMvigor210CoreBiologies.tar.gz. Code for data processing, analysis and plotting and the R script are available from this IMvigor210CoreBiologies package.

The IMvigor210 study was a phase 2, multicenter, single-arm, open-label, and 2 cohort trial that assessed atezolizumab as a treatment for metastatic urothelial cancer in cisplatin-ineligible patients [25]. Clinical data for the first-line cisplatin-ineligible IMvigor210 cohort was collected from 47 academic medical centers and community oncology practices across 7 countries in North America and Europe. All participants in the study consented.

The IMvigor210 dataset includes recorded responses to immune checkpoint blockade. This Illumina HiSeq 2500-based dataset contains 348 subjects (76 female and 272 male) with 17692 gene expression biomarkers (i.e., features), which were derived from genes using Entrez gene ID and gene symbol. Archival tumor tissues were collected for biomarker assessments, and gene expression was designed to be quantified for a T-effector gene signature (consisting of CD8A, GZMA, GZMB, PRF1, INFG, and TBX21) [5]. The feature values of gene information were normalized using the trimmed mean of M-values (TMM) method. Each sample includes corresponding clinical labels, such as age, sex, PD-L1 status of immune cells, prior tobacco use, metastatic disease, best confirmed overall survival, overall response, Response Evaluation Criteria in Solid Tumor (RECIST), immune phenotype, and The Cancer Genome Atlas (TCGA) subtype. For this study, three specific immune phenotypes were investigated: immune deserts, immune-excluded, and inflamed.

All types of human cancers, including BC, can be categorized into three immune phenotypes. These phenotypes are distinguished by the strength and relationship of the immune response of T-cells acting on the tumors, and different treatments should be applied based on the individual immunological biology of each phenotype. The IMvigor210 dataset consists of 76, 134, and 74 samples of immune deserts, immune-excluded, and inflamed phenotypes, respectively. The immune desert subtype is absent of immune cells, with total lack of an immune response against the tumor. The immune-excluded subtype has an immune response with only peripheral invasion of T-cells that cannot completely overwhelm the tumor. The inflamed subtype involves an active immune response where inflammatory myeloid cells and activated CD8+ T-cells exist in the tumor [26], [27]. Since the remaining 64 samples in the dataset did not provide any information on immune phenotypes, they were disregarded for this study.

### C. Classification Method

Five powerful ML-based classification algorithms, i.e., Support Vector Machine (SVM), Random Forest, XGBoost, AdaBoost and deep neural network (DNN) were adopted to investigate immune phenotypes using gene expression features [28]–[32]. We performed a supervised learning task, where each data sample consists of a feature vector and class label. In our experiment, the algorithms were trained to learn optimized mapping between the features (i.e., gene expression) and target labels (i.e., immune phenotypes).

SVM is a well-known supervised classification algorithm that can learn a decision boundary, either linear or non-linear, in a feature space. Given data samples forming individual clusters in the feature space according to class labels, SVM learns a decision boundary that maximizes the margin of distance between the decision boundary and other clusters [33]. Such a criteria intuitively makes sense as the distance between individual clusters and the learned decision boundary will be balanced. To train a linear model when the data are not linearly separable, the model requires a regularizer with a user parameter (i.e., slack variable) that controls the margin and tolerable error within the margin. Training a non-linear model requires a kernel function (e.g., Gaussian and polynomial kernels) that can map the data onto a high-dimensional space where the data can become linearly separable. Taking the trained decision boundary back to the original space will then yield an optimized non-linear decision boundary [34].

Random Forest is one of the ensemble methods for classification and regression tasks. A sole Decision Tree can perform the same tasks on supervised learning problems by asking a series of questions regarding to the characteristics of input variables. To avoid overfitting with large trees [35], [36], Random Forest incorporates multiple Decision Trees and casts a majority vote from the results classified from each tree. This ensemble technique is known as Bagging [37], which is an abbreviation of Bootstrap Aggregation. It is a method of extracting samples multiple times (Bootstrapping [38]) and training each model to aggregate the results. Although some trees created by Random Forest can be overfitted, an overwhelming majority can suppress the flaw from having a significant impact on prediction of class labels, i.e., classification.

In addition, we adopted another ensemble method, Boosting algorithm [39], based on the Decision Tree architecture. Unlike to Bagging where each tree makes independent decisions, Boosting has a sequential prediction process in which one model influences the decision of the next tree. In this process, Boosting repeats multiple steps to create a new classification criterion by improving weights on misclassified data. Finally, it creates a strong classifier gathering weak classifiers altogether to result in the ensembled output. In this paper, we used XGBoost [40] and Adaptive Boost (AdaBoost) [41], [42]. The difference of two methods is the way to deliver information of misclassified data from previous models. For example, AdaBoost updates subsequent classifiers based on the weight values of the former models. However, the update of XGBoost is based on gradient descent with a greedy algorithm.

Lastly, for the deep learning (DL) approach, we used a DNN algorithm with multiple hidden layers [30]. This consisted of an input layer for the original data, output layer for prediction outcome (e.g., pseudo-probability for each class), and a varying number of hidden layers where the input data can be transformed and model parameters are trained to minimize prediction error, usually defined by cross-entropy. While the input and output layers contain nodes according to the input dimension and the number of class labels respectively, each hidden layer is composed of hidden nodes determined by a user. At each hidden node, the node from its previous layer becomes the input, which is connected to the hidden node via edges with corresponding edge weights. The input values and edge weights at each hidden node are first linearly combined and then fed into a non-linear activation function (e.g., sigmoid or rectified linear unit (ReLU)) to yield an output that goes into the following layer as an input. At the output layer, the outcome values from each node are normalized to yield a pseudo-probability that tells which class label is the most likely for a given data sample. The l1- and l2-regularizers were applied onto the model parameters for sparsity as in least absolute shrinkage and selection operator (LASSO [43], [44], depicting important features only by suppressing weights of unimportant features to 0) and to make the model stable [28].

### D. Model Training

In order to obtain unbiased results, we used 10-fold cross validation (CV) to conduct experiments with the two five classification algorithms [45]. For the SVM, we utilized both linear and non-linear models. An RBF kernel was used for the non-linear classifier. The slack variable C was varied from 0.01 to 1000 to find the best performance. For Decision Tree-based models, such as Random Forest, XGBoost and AdaBoost, the number of trees per fold was kept to the same rate for comparing all results under unbiased conditions. The number of Decision Trees per fold was set to 100 and all Decision Trees were generated by allowing random sampling with replacement. The final classification was decided by majority voting incorporating outputs from every single classifier. The number of Decision Trees in all Boosting methods was set to 100. As for learning rates, XGBoost and AdaBoost were set to 0.1 and 1.0 respectively, with the highest test accuracy score for each classifier. For the DNN, we tried multiple settings by adjusting the number of hidden layers, nodes, and regularizers. The number of hidden layers varied from 0 to 3, and the number of hidden nodes in each layer varied between 16 and 1024. A drop rate ranging from 0.1 to 0.5 was applied. To measure the error of the model, cross-entropy was used. For the activation function, ReLU was used and Softmax was applied at the output layer to obtain the likelihood for each class. The overall model was trained by backpropagating the error from cross-entropy with gradient descent using the Adaptive Moment Estimation (Adam) optimizer [46].

### E. Feature Selection

Since the data is compiled in a very high-dimensional space, statistical hypothesis tests were used to select effective features for distinguishing different groups. Statistical group analysis for each pair of phenotypes was applied on each feature, and resultant p-values were corrected for multiple comparisons using Bonferroni correction at the 0.05 confidence level. The feature selection process was applied only at the training stages (i.e., excluding test data) across each fold in CV where the phenotype labels were available; hence, avoiding circular analysis.

### F. Evaluation

To evaluate the performance of our classification results, we measured accuracy, precision, and recall. Accuracy was computed as the ratio of the number of correct predictions out of the total number of samples in a testing dataset. Precision and recall were considered for binary classification (i.e., positive vs. negative); precision measures how precise the prediction is for the positive class, while recall measures how much of the positive samples in the training dataset are correctly covered by the prediction. While accuracy is an intuitive and important measure for evaluation, precision and recall are also important for evaluating data with imbalanced class labels. Since precision and recall are computed for binary classification tasks, we computed them in a one-versus-all manner; out of the three immune phenotype classes, one of them is selected as positive. The other two were combined and considered the negative class. This is iterated for all the three classes as positive, yielding three individual results. We also plotted receiver operating characteristic (ROC) and precision and recall (PR) curves. The area under the curve (AUC) was computed for evaluation (higher AUC denotes better performance). To understand the effectiveness of a classifier on an imbalanced dataset, the AUC scores of both curves were used as quantified summaries of the model performance as well as Mathews Correlation Coefficient (MCC) at a threshold of 0.5 to determine positive and negative labels. These values ranged between 0.0 to 1.0, with larger scores suggesting that a model is more robust.

### G. Implementation Environment

All experiments were implemented in Python on a Nvidia GeForce RTX 2070 SUPER graphic card. DNN was designed based on Keras and scikit-learn machine learning libraries were utilized for the other methods. As for statistical tests, scipy library was used to derive p-values.

## III. Results

Classification results on Immune Phenotypes of BC using the five classification methods are demonstrated in this section.

### A. Classification of Immune Phenotypes With SVM

Immune phenotyping of BC from the Imvigor210 dataset resulted in three subtypes, inflamed, immune-excluded, and immune desert; all of which are characterized by distinct T lymphocyte infiltration patterns. Immune desert tumors have

Evaluation measures were averaged across 10-fold. These values range between 0 and 1, with values closer to 1 indicating better performance. The area under the curve (AUC) of precision and recall (PR) curves accounts for the class imbalance in performance evaluation poor infiltration of immune cells (absence of pre-existing antitumor immunity), immune-excluded tumors only exhibit retention of T lymphocytes in the reactive stroma, and inflamed tumors show infiltrated T lymphocytes [47], [48]. The overall results are summarized in Table 1.

**TABLE 1.** Comparison of Representative Results in Different Settings

Classifier	Mean Train Acc	Mean Test Acc	MCC (Threshold: 0.5)	Mean Precision (std)	Mean Recall (std)	Mean AUC of PR (std)	Mean AUC of ROC (std)
SVM (Linear)	1	0.655	0.457	0.667 (0.036)	0.645 (0.048)	0.674 (0.031)	0.799 (0.083)
SVM (RBF)	1	0.655	0.456	0.655 (0.018)	0.644 (0.048)	0.678 (0.034)	0.798 (0.083)
SVM (RBF) (feature selection)	0.963	0.68	0.495	0.701 (0.035)	0.663 (0.118)	0.688 (0.034)	0.822 (0.066)
Random Forest	1	0.496	0.344	0.752 (0.099)	0.473 (0.104)	0.670 (0.045)	0.795 (0.086)
Random Forest (feature selection)	1	0.570	0.404	0.737 (0.089)	0.558 (0.051)	0.691 (0.051)	0.817 (0.077)
XGBoost	1	0.623	0.406	0.647 (0.043)	0.606 (0.103)	0.646 (0.073)	0.793 (0.081)
XGBoost (feature selection)	1	0.605	0.380	0.623 (0.029)	0.598 (0.059)	0.616 (0.055)	0.770 (0.089)
AdaBoost	0.821	0.613	0.386	0.714 (0.144)	0.547 (0.287)	0.649 (0.094)	0.776 (0.135)
AdaBoost (feature selection)	0.810	0.577	0.314	0.632 (0.116)	0.528 (0.239)	0.576 (0.110)	0.726 (0.163)
DNN (feature selection)	0.715	0.641	0.473	0.679 (0.045)	0.626 (0.101)	0.755 (0.099)	0.875 (0.054)
DNN (l1 regularizer)	0.834	0.616	0.412	0.685 (0.069)	0.590 (0.109)	0.771 (0.118)	0.870 (0.027)
DNN (feature selection, l1 regularizer)	0.719	0.666	0.488	0.722 (0.084)	0.635 (0.134)	0.771 (0.092)	0.860 (0.039)

The classification process using an SVM-based system was implemented with two types of kernel functions (i.e., linear kernel and radical basis function (RBF)). As shown in Table 1, the best accuracy scores of both SVM experiments without feature selection were 0.655 while their training accuracies were 1. This indicates that there was a serious overfitting (i.e., the model worked perfectly on the training data but significantly failed to do so for testing data). The slack variable utilized in the two cases were 100. When statistical feature selection was applied to the input data of SVM with RBF kernel, the average test accuracy across CV scored the highest (0.68) throughout all experiments, which suggests that feature selection based on statistical group tests was effective. For slack variables, the score reached a peak at 10 and decreased slightly as the variables changed. On the other hand, linear SVM with feature selection yielded poor results. The accuracy was 0.588 regardless of the slack variable.

**Figure 1.**
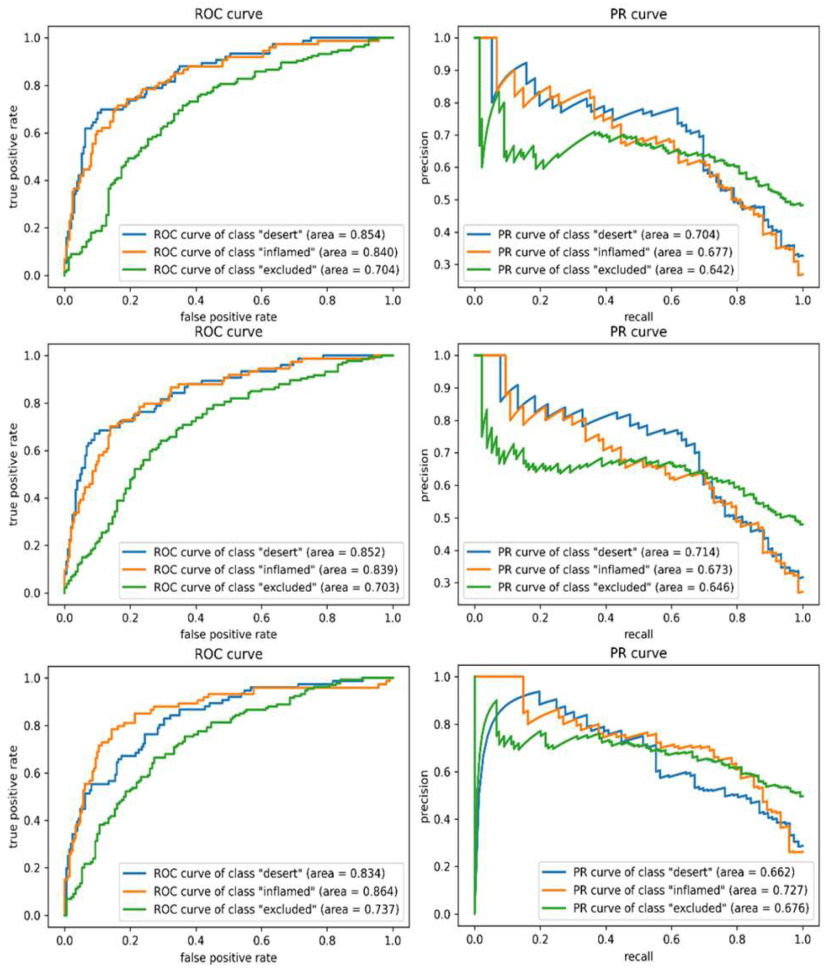
**Receiver operator characteristic (ROC) and precision and recall (PR) curves for each class using support vector machine (SVM)**. Top: Linear SVM, mid: SVM (RBF), bottom: SVM (RBF) with feature selection. Higher AUCs, closer to 1, indicate better performance. High AUCs with ROC curves for each phenotype indicate the model is predicting the phenotypes with low false positives. PR curves show that classification performance for the immune-excluded class is enhanced (green line) by feature selection.

In Fig. 1, PR and ROC curves for the three SVM experiments are described for the 3 classes, which are marked in blue (immune desert), orange (inflamed), and green (immune-excluded). Among the results with various SVMs, similar to the results of the test accuracy, SVM with RBF kernel and feature selection resulted in the highest average

**Figure 2.**
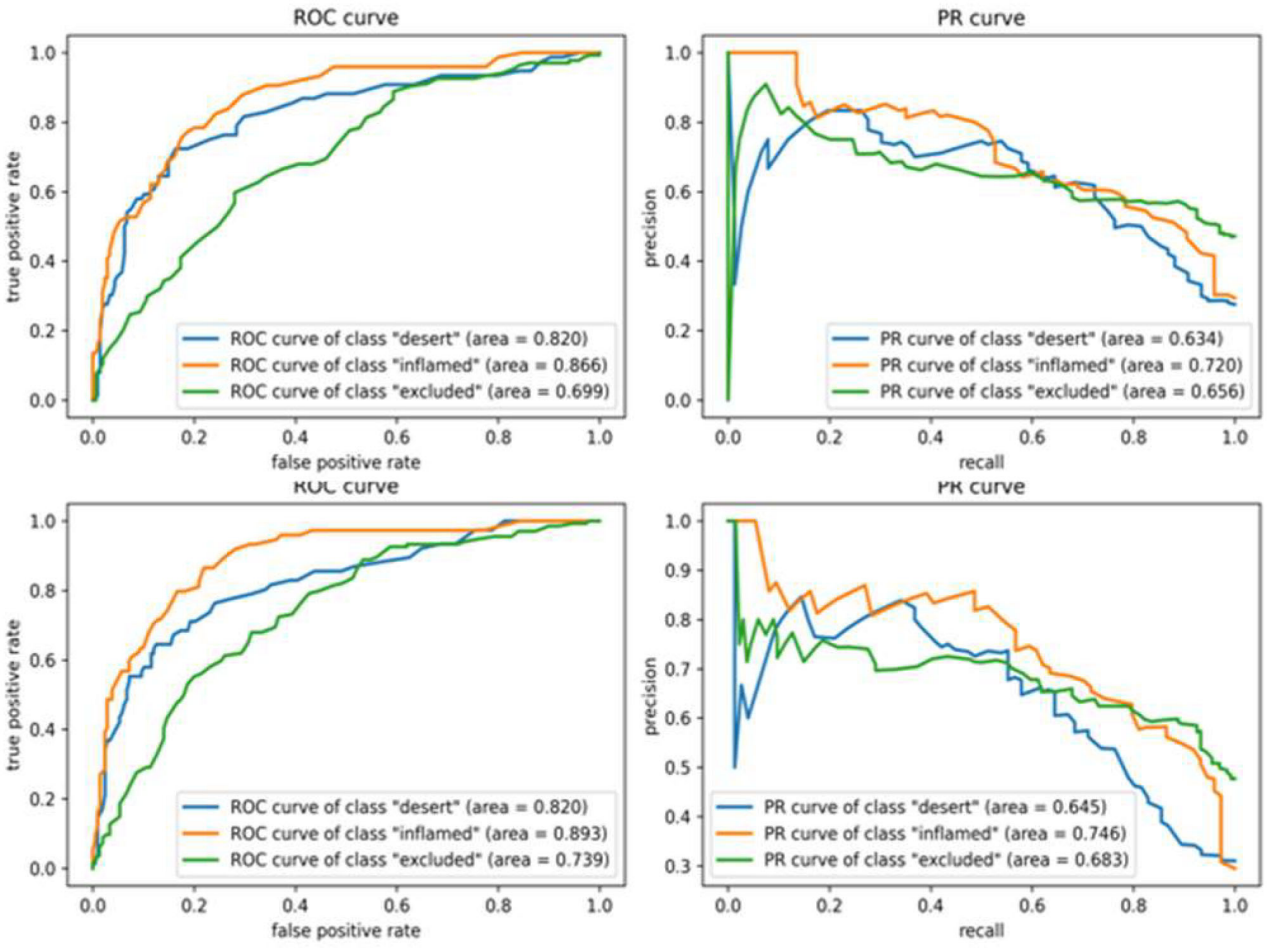
**Receiver operator characteristic (ROC) and precision and recall (PR) curves for each class using Random Forest**. Top: Random Forest without feature selection, bottom: Random Forest with feature selection. Higher AUCs, closer to 1, indicate better performance. High AUCs with ROC curves for each phenotype indicate the model is predicting the phenotypes with low false positives. ROC curves show that classification performance for the immune-excluded class is enhanced (green line) by feature selection. Likewise, comparing two PR curve plots illustrates that performance of all classes with feature selection has outperformed.

**Figure 3.**
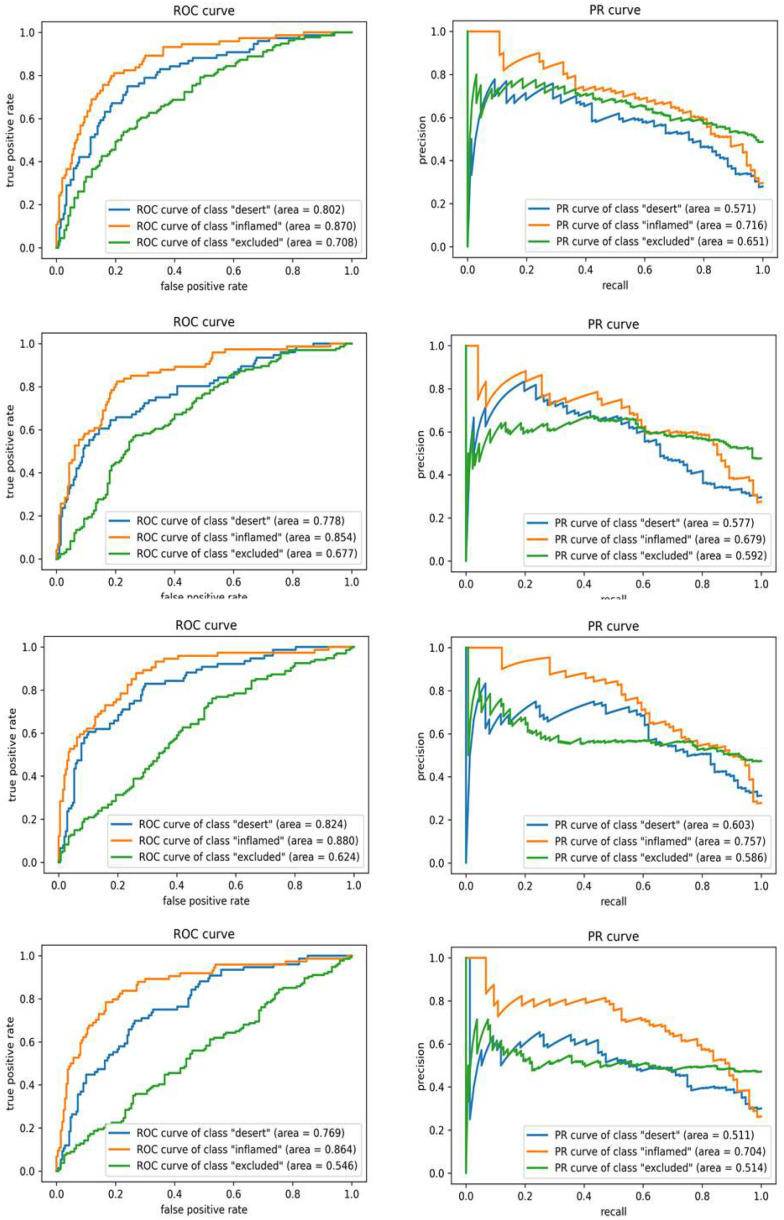
**Receiver operator characteristic (ROC) and precision and recall (PR) curves for each class using XGBoost and AdaBoost**. Top: XGBoost without feature selection, second row: XGBoost with feature selection, third row: AdaBoost without feature selection, bottom: AdaBoost with feature selection. Higher AUCs, closer to 1, indicate better performance. High AUCs with ROC curves for each phenotype indicate the model is predicting the phenotypes with low false positives. Almost all classes of both Boosting algorithms without feature selection shows better AUCs of PR and ROC curves than feature selected models.

AUC scores for both metrics among SVM results; 0.688 and 0.812 for the PR and ROC curves, respectively. Accordingly, MCC of 0.495 for this case was the highest as well. Notably, all of the averaged AUC scores of the PR and ROC curves across SVM classes were recorded slightly smaller than the results from DNN models.

### B. Classification of Immune Phenotypes With Random Forest

Test accuracy of Random Forest scored the lowest throughout all experiments regardless of feature selection. Similar to SVM, training accuracies of Random Forest were 1, denoting that this algorithm has also overfitted to the input data and yielded poor test accuracy and MCC. But interestingly, we can see that mean precision recorded the highest score among all models as shown in Table 1, whether feature selection is applied or not. This highest precision value indicates that Random Forest was able to produce the lowest number of false positive samples. Notably, applying Bonferroni correction reduced the gap between precision and recall, so that the AUC scores of all classes in PR and ROC plot (shown in Fig. 2) outperformed to those of non-feature selected Random Forest.

### C. Classification of Immune Phenotypes With Xgboost and Adaboost

For Boosting methods, the most representative Boosting algorithms, AdaBoost and XGBoost were employed and their performance curves are shown in Fig. 3. As shown in Table 1, the overall test accuracy and MCC of both Boosting algorithms scored higher than Random Forest but lower than SVM and DNN. Although XGBoost was overfitted for training data, on the contrary to AdaBoost, the test accuracy of XGBoost was slightly higher than for AdaBoost's. Also, applying feature selection to Boosting classifiers resulted a worse performance for all metrics compared to models without Bonferroni correction.

Therefore, we can see that the feature selection was invalid in respect of Boosting algorithms that focus weights on misclassified samples for improving accuracies. In other words, the eliminated features from Bonferroni correction have had a substantial influence on decision-making processes in Boosting models, especially for identifying the attributes of incorrectly classified dataset.

### D. Classification of Immune Phenotypes With DNN

Various classification experiments using DNN were performed with the settings described in the Methods section. Representative results are summarized in Table 1. With a very naïve DNN model without any regularizers or techniques to make the model robust (i.e., dropout, batch normalization, and feature selection), the resultant accuracy averaged across all 10 folds was 0.549. Considering the baselines with random guess (0.33) and prediction as the dominant class (0.472), the model was properly learning to predict BC immune phenotypes. However, it suffered from overfitting and relatively low accuracy compared to the SVM-based models. Applying a dropout rate of 0.3, statistical feature selection, and l1-regularizer (with hyper parameters 0.01 and 0.08 for each layer) on two hidden layers with 32 hidden nodes, the accuracy increased to 0.666 with averaged respective precision and recall of 0.722 and 0.635 across different class labels.

**Figure 4.**
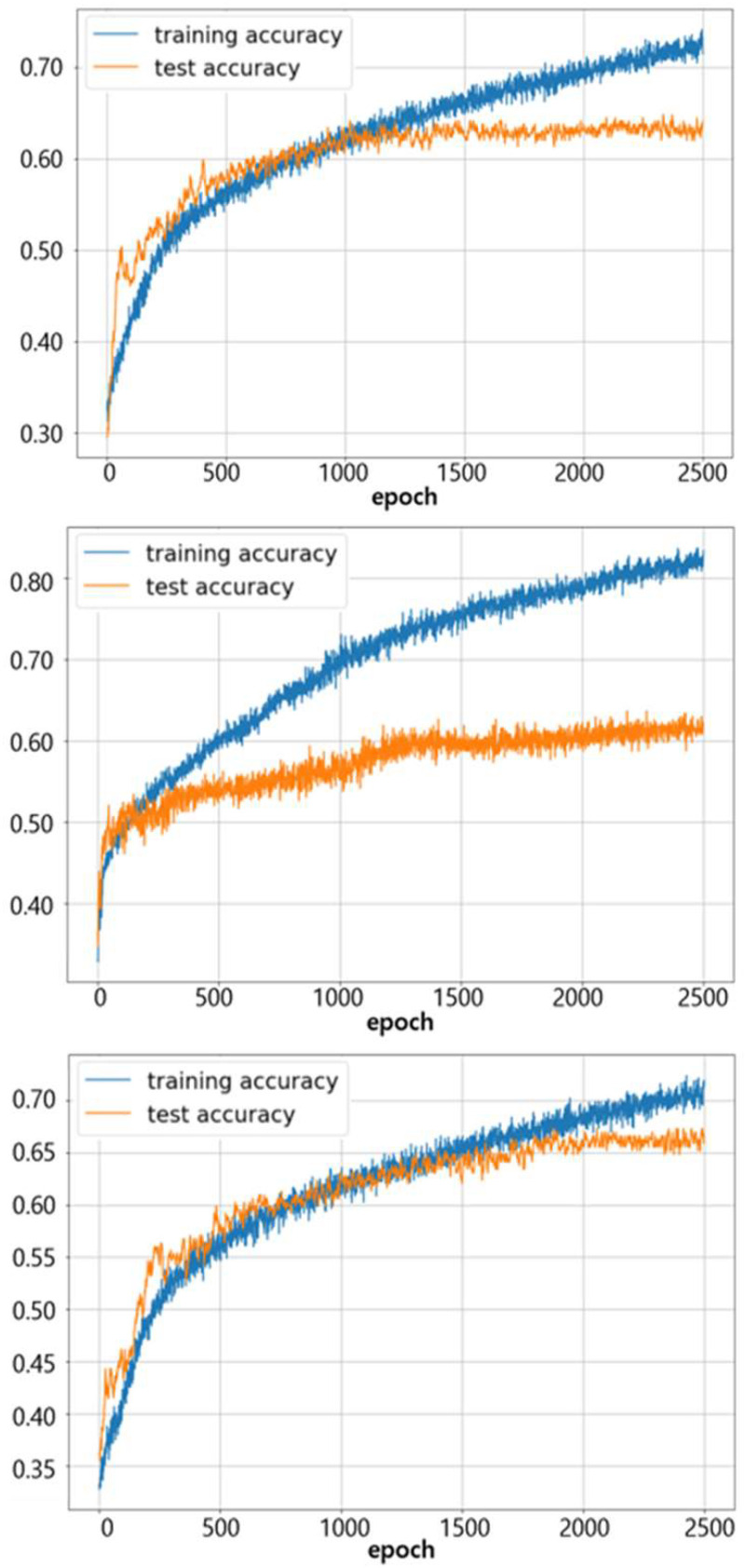
**Change of training / testing accuracy with respect to epoch in DNN training**. Top: DNN (feature selection), middle: DNN (L1-norm penalty), Bottom: DNN (L1-norm penalty and feature selection). Similar training (blue) and testing (orange) accuracies indicate better generalization of the trained model to unseen testing data. As seen in the middle panel, significant overfitting (large differences between training and testing accuracies) occurs without feature selection.

**Figure 5.**
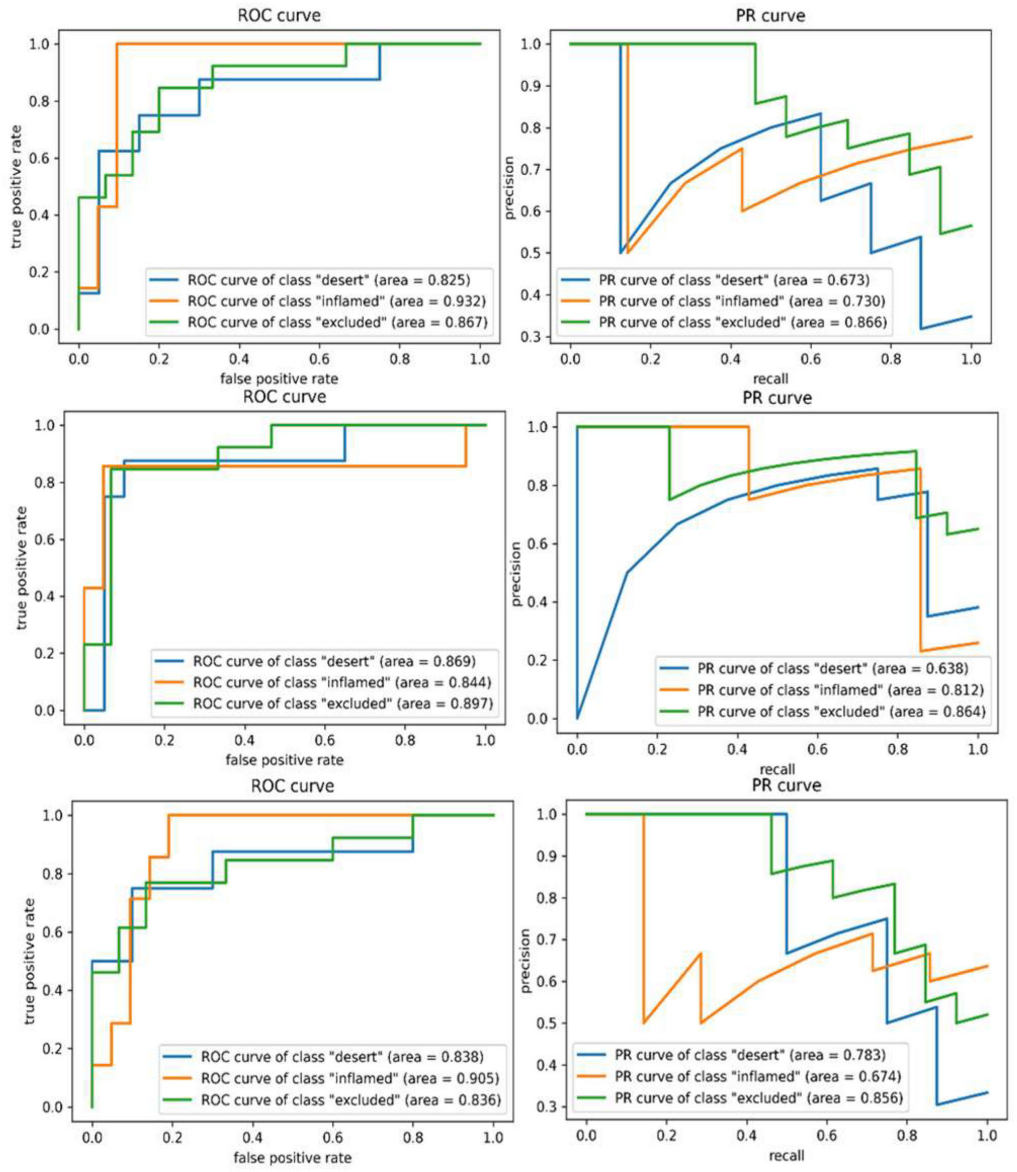
**ROC and PR curves (for each class) using deep neural network (DNN)**. Top: DNN (feature selection), mid: DNN (L1-normpenalty), bottom: DNN (L1-norm penalty and feature selection). Higher AUC (closer to 1) indicates better performance. High AUCs with ROC curves for each phenotype indicate the model is predicting the phenotypes with low false positives. Overall AUCs of both ROC and PR curves are higher than those from SVM analysis.

The ROC and PR curves for individual experiments are shown in Fig. 4, where the curves for each class are given in blue (immune desert), orange (inflamed), and green (immune-excluded). All the ROC curves in the left column of Fig. 4 rapidly converged close to 1 in their true positive rate (TPR). Simultaneously, the PR curves in the right column of Fig. 4 maintained precision with respect to recall as much as possible. The respective AUCs of 0.77 and 0.87 for the PR and ROC curves demonstrated the model's feasibility in classifying different stages of immune phenotypes. The training and testing accuracies for different DNN settings are shown in Fig. 5, which demonstrates that both training (blue) and testing (orange) accuracies increase as the training progresses. After the model convergences, the middle subfigure (with l1-penalty) shows a large difference between the training and testing accuracies as opposed to the other two subfigures. These differences were due to the application of statistical feature selection using a t-test. For each fold, statistical testing at each gene feature on the training data with Bonferroni correction at 0.05 yielded 900∼1300 significant features. Given the high dimensionality of the data, without feature selection for dimension reduction, the issue of overfitting was easily seen. Although not presented in these results, we also observed overfitting occurring with an increase in hidden layers or nodes. This overfitting behavior explains the differences in MCC. As seen in Table 1, the DNN with l1-penalty only showed the lowest MCC as it was highly overfitted. On the other hand, the DNN with both l1-penalty and feature selection did not overfit and demonstrated the highest MCC of 0.488.

With the l1-regularizer at imposing sparsity at the input layer, many of the weights associated with each feature were suppressed to a value of or close to 0. From the DNN model with regularizer and feature selection, which yielded the highest accuracy and AUC for PR curves, the top 20 highest weighted gene features across all 10 folds were identified. Among them, 13 common features existed across all folds. These were named TMEM156 (Transmembrane Protein 156), TOX (Thymocyte Selection-associated High-mobility Group Box Protein), XAF1 (X-linked Inhibitor of Apoptosis-associated Factor-1), SPATC1 (Spermatogenesis and Centriole Associated 1), FOXP3 (Forehead Box P3), ARRB2 (Arestin Beta 2), TNFRSF9 (TNF Receptor Superfamily RNASE6 (Ribonuclease A Family Member K6), DBH-AS1 (DBH Antisense RNA 1), TENT5C (Terminal Nucleotidyltransferase 5C), ID3 (DNA-binding Protein Inhibitor), APOE (Apolipoprotein E), and LAX1 (Lymphocyte Transmembrane Adaptor 1).

## IV. Discussion

In recent years, immunotherapy has come to play an increasingly important role in oncology. Immunotherapy in cancer treatment involves modifying or adding defense mechanisms to the patient's immune system. Immunotherapy is often used as a supplement to conventional cancer treatment methods, such as surgery, chemotherapy, and radiation therapy. For some specific types of lung and colorectal cancer, immunotherapy is used as the first line of treatment [49]. In urological oncology specifically, immunotherapy is used as a supplemental treatment in addition to standard of care [50]. Immunotherapy in cancer treatment involves modifying or adding defense mechanisms to the patient's immune system. Currently, immunotherapy can be divided into several types, including immune CPIs, T cell transfer therapy, monoclonal antibodies, therapeutic vaccines, and immune system modulators [51].

Based on current research on BC therapies, immunotherapy seems to be the most promising. Because there are multiple regimens for immunotherapy, patients respond differently depending on the therapy. Currently, the US FDA has approved five anti-programmed death-1/ligand 1 (PD-1/L1) checkpoint inhibitors: atezolizumab, avelumab, durvalumab, nivolumab, and pembrolizumab [52]. Among them, atezolizumab was the first to pass approval. This approval was made based on the research results of IMvigor210. IMvigor210 was an open multicenter, single-arm phase II clinical study designed to study whether atezolizumab could become the standard treatment for advanced urothelial cancer. This study suggested that for patients with platinum-based refractory metastatic urothelial carcinoma (mUC), checkpoint inhibitors seem to be more attractive than chemotherapy [13]. Atezolizumab has shown encouraging long-term response rates, survival rates, and tolerability, supporting its therapeutic use in untreated mUC [53]. Based on the results of the study, the FDA approved atezolizumab as the first-line drug for the treatment of patients with advanced urothelial cancer who are not suitable for cisplatin chemotherapy.

Regarding Boosting methods, the key hyperparameters were the number of trees and learning rates. The number of estimators designates the scale of Random Forest. As more individual trees were included, the classification performance became better, but the whole model took longer time to be trained. A learning rate of Boosting algorithms denotes a coefficient applied to the weak classifiers when calibrating the error values sequentially. Since the learning rate directly affects the variation in the weight update, the difference in decision boundaries of multiple trees changed proportionally to the learning rate. However, it requires a large number of trees with a time-consuming ensemble process at the same time. Thus, the number of trees and learning rate has a trade-off relationship and coordinating the ratio between the two parameters was crucial to the performance of classification. Therefore, we had to manage the number of estimators at the same rate for fair comparison of the results.

In our study, Decision Tree based methods mostly tended to overfit as the training accuracies reached 1, and testing cases underperformed compared to SVM and DNN. Comparing the top-2 algorithms, although the accuracies with our DNN model were lower than that of our SVM model, the AUCs of evaluation curves (ROC and PR) were better. Specifically, the AUCs of PR curves in the DNN model were larger by 0.083 compared to the best of both models, which demonstrates that the DNN did better with imbalanced class labels. This is because the latent space for group separation found by DNN is better than SVM; while SVM with RBF kernel maps the data onto a higher dimensional space to find a linear decision boundary in that space, the DNN model mapped data onto a lower-dimensional space where group separation can be more effective and robust. The accuracy may be better in the high-dimensional space found by SVM with RBF kernel, but the actual separation of the three immune phenotypes was more effective with DNN. This was also seen in the overfitting trend of both models. Both SVM and DNN suffered from overfitting; it was more serious for the SVM model while the DNN model was able to mitigate this issue with common techniques, such as dropout and regularizers, and this behavior was observed in MCC of individual models. As a result, there was a trade-off between training accuracy and other measures. Although SVM achieved slightly better test accuracy and MCC than those from DNNs, the precision and AUCs were significantly higher in DNN models, which we believe are more important.

Regarding the effective biomarkers found by the DNN model, downstream statistical group tests across each phenotype pairs yielded many significant p-values. As the phenotype profiles are ordered by severity, all 13 features showed very low p-values (<1e-6) for immune desert vs. inflamed and mostly effective (i.e., <0.05) for other group pairs. Perhaps this was expected as our feature selection process selected important features with statistical tests at the training stage, but it was still worth analyzing them over the entire data to confirm if these biomarkers are really statistically meaningful for group comparisons.

We further investigated the 13 significant features associated with immunotherapy responsiveness in BC. FOXP3 is widely known as a key regulatory transcription factor of regulatory T cells, contributing to immune system responses [27], [54], [55]. Expression of FOXP3 in BC has been reported to negatively associated with survival of patients [56]. Recent studies have reported that FOXP3 acts as a transcriptional regulator of HIF-1α gene expression in BC, suggesting the potential contribution of the FOXP3/HIF-1α pathway in poorer survival [57]. APOE, an apolipoprotein related to lipoprotein-mediated lipid transport, was also found in the immunotherapy responsive molecular features. The LXR (liver X receptor)/APOE axis has been reported to regulate innate immune suppression and activation. Since this axis blocks innate immune suppression in many cancer types, it has been suggested as a therapeutic target to allow better efficacy of immunotherapy for cancer patients [58]. TOX has been found to regulate innate immunity and the tumor microenvironment. TOX expression significantly increases immune infiltration levels and is downregulated in most cancer types. Lower expression of TOX is correlated with poorer prognoses, suggesting that TOX expression can be used for stratification of non-responders to immunotherapy [59], [60].

Findings from this study suggest that the experiment we designed using ML algorithms are effective in classifying immune phenotypes of BC with gene expressions and identifying associations between specific gene expressions and the phenotypes. It also demonstrates the potential of our DNN model after improving overfitting via utilization of more samples. In addition, this study found 13 features associated with response to immunotherapy, which may all be biologically relevant.
